# *Mycobacterium avium* Subspecies *paratuberculosis* Infection Modifies Gut Microbiota under Different Dietary Conditions in a Rabbit Model

**DOI:** 10.3389/fmicb.2016.00446

**Published:** 2016-03-31

**Authors:** Rakel Arrazuria, Natalia Elguezabal, Ramon A. Juste, Hooman Derakhshani, Ehsan Khafipour

**Affiliations:** ^1^Department of Animal Health, NEIKER-Instituto Vasco de Investigación y Desarrollo AgrarioDerio, Spain; ^2^Department of Animal Science, University of Manitoba, WinnipegMB, Canada; ^3^Department of Medical Microbiology, University of Manitoba, WinnipegMB, Canada

**Keywords:** *Mycobacterium avium* subsp. *paratuberculosis*, rabbit, animal model, high fiber diet, gut microbiota, inflammation

## Abstract

*Mycobacterium avium* subspecies *paratuberculosis* (MAP) the causative agent of paratuberculosis, produces a chronic granulomatous inflammation of the gastrointestinal tract of ruminants. It has been recently suggested that MAP infection may be associated with dysbiosis of intestinal microbiota in ruminants. Since diet is one of the key factors affecting the balance of microbial populations in the digestive tract, we intended to evaluate the effect of MAP infection in a rabbit model fed a regular or high fiber diet during challenge. The composition of microbiota of the cecal content and the sacculus rotundus was studied in 20 New Zealand white female rabbits. The extracted DNA was subjected to paired-end Illumina sequencing of the V3-V4 hypervariable region of the 16S rRNA gene for microbiota analysis. Microbial richness (Chao1) in the cecal content was significantly increased by MAP infection in regular diet rabbits (*p* = 0.0043) and marginally increased (*p* = 0.0503) in the high fiber group. Analysis of beta-diversity showed that MAP infection produces deeper changes in the microbiota of sacculus rotundus than in the cecal content. A lower abundance of Proteobacteria in the cecal content of infected animals fed the high fiber diet and also lower abundance of Bacteroidetes in the sacculus rotundus of infected animals fed the regular diet were observed. Based on OPLS-DA analysis, we observed that some bacteria repeatedly appear to be positively associated with infection in different samples under different diets (families Dehalobacteriaceae, Coriobacteriaceae, and Mogibacteriaceae; genus *Anaerofustis*). The same phenomenon was observed with some of the bacteria negatively associated with MAP infection (genera *Anaerostipes* and *Coprobacillus*). However, other groups of bacteria (Enterobacteriaceae family and ML615J-28 order) were positively associated with infection in some circumstances and negatively associated with infection in others. Data demonstrate that MAP infection and diet changes do interact and result in shifts in the microbiota of the cecal content and sacculus rotundus of rabbits.

## Introduction

*Mycobacterium avium* subspecies *paratuberculosis* (MAP) is the causative agent of paratuberculosis (PTB) a chronic granulomatous inflammation of the gastrointestinal (GI) tract of ruminants. The infection may be cleared by the host or develop into subclinical or clinical disease causing significant economic losses in livestock (Clarke, 1997; Kennedy and Benedictus, 2001).

Although PTB has been historically linked to domestic ruminants, wild-life ruminant, and non-ruminant species are also susceptible to infection (Chiodini and Van Kruiningen, 1983; Beard et al., 2001a; Forde et al., 2013). In addition, association of MAP with Crohn’s disease (CD; Naser et al., 2004; Juste et al., 2008) and Type 1 diabetes (Masala et al., 2011; Naser et al., 2013) in humans adds further interest to the role of this disease as a potential zoonosis.

It is known that the gut microbiome plays an important role in competitive exclusion of pathogens and in development and maturation of intestinal mucosal immunity (Kau et al., 2011; Stecher and Hardt, 2011). Many studies have documented differences in the composition of host associated microbial communities between healthy and diseased states (Clemente et al., 2012; Karlsson et al., 2013; Knights et al., 2013). It is recognized that an altered microbiome is not just a marker of disease but that it also actively contributes to pathogenesis (Chassaing et al., 2012). Reduced diversity of both fecal and mucosa-associated microbiota has been extensively reported in patients suffering from chronic intestinal inflammatory disease (Manichanh et al., 2006; Dicksved et al., 2008; Walker et al., 2011). Moreover, the composition of gut microbial communities may also modulate Type 1 diabetes (Wen et al., 2008).

Additionally, it is important to note that diet is one of the key factors affecting the balance of microbial populations in the digestive tract (Xu and Knight, 2014; Graf et al., 2015). Dietary nutrients are the principal substrates for the microbial population and also have a direct effect on the immune response (Faria et al., 2013). In laboratory mice, dietary-mediated effects are evident in a GI infection model (Ooi et al., 2014). Although the diet role in MAP infection has been scarcely studied, in previous experiments we observed that short term dietary shifts can modulate PTB infection in a rabbit model, showing differences in histopathological lesion extension and bacterial load on gut associated lymphoid tissue (GALT; Arrazuria et al., 2015a).

Rabbits are naturally susceptible to the development of MAP infections causing severe PTB-associated inflammatory responses in its wild habitats (Beard et al., 2001b; Maio et al., 2011), and milder pathological changes under experimental conditions (Vaughan et al., 2005; Arrazuria et al., 2015a). These characteristics, together with their size and handling easiness make rabbits a convenient experimental species to study chronic intestinal diseases of animals and humans. One specific feature of rabbit GALT is that it is composed of two additional special structures: the sacculus rotundus, which is located at the ileo-cecal junction, and the vermiform appendix, located at the end of the cecum. These two lymphoid organs account for more than 50% of the total lymphoid tissue in the rabbit (Rees Davies et al., 2003). These tissues can induce an effective immune response and it has been demonstrated that a specific microbial composition on the vermiform appendix can induce inflammation (Shanmugam et al., 2005). The sacculus rotundus closely resembles the ileocecal-valve in ruminants, and in previous MAP infection studies with rabbits we detected higher MAP loads in sacculus rotundus compared to vermiform appendix and any other sites in the intestine (Arrazuria et al., 2015a).

Recent advances in next generation sequencing technologies have enabled researchers to investigate the complete microbial composition of the GI tract under different conditions (Schuster, 2008). In the present study, high-throughput sequencing of the 16S rRNA gene was performed to determine MAP infection associated shifts in the microbial composition of cecal content and sacculus rotundus.

Previous results from our group have shown that diet shifts can modulate MAP infection (Arrazuria et al., 2015a). In humans, high fiber diets have been shown to promote and increase the gut microbiota diversity (Steinle et al., 2013) and also to diminish inflammatory responses by a mechanism that includes shaping the intestinal microbiome and indirectly affecting the immune system (Kuo, 2013). On the basis of previously obtained results, in which a high fiber diet during challenge produced MAP infection modulation (Arrazuria et al., 2015a), we again performed this dietary shift to investigate if it produces changes in gut microbiota that could help understanding MAP infection modulation.

We hypothesized that as in other chronic intestinal diseases, MAP infection may produce changes in the gut microbiota. Therefore, our aim was to evaluate changes in gut microbiota in rabbits challenged with MAP and fed either a regular or a high fiber diet.

## Materials and Methods

### Animals and Experimental Designs

**FIGURE 1:**
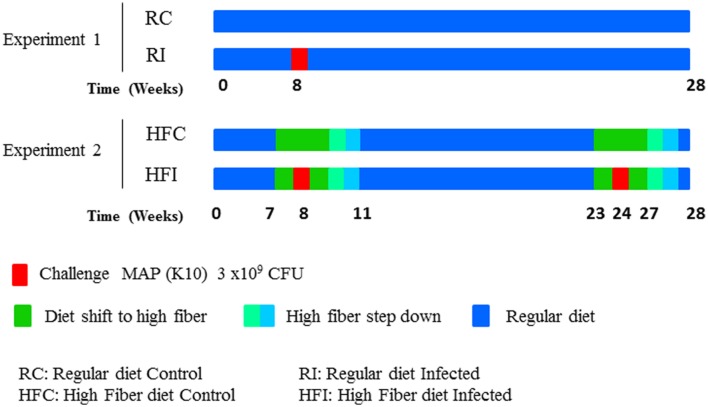
**Experimental design diagram**.

New Zealand white female rabbits (*n* = 20) were purchased from authorized experimental animals dealers, Granja San Bernardo, Tulebras, Spain and arrived at NEIKER animal facilities, Derio, Spain, at the age of 6 weeks and 1.5 kg of body weight. After a 2-week adaptation period during which rabbits were fed special weaning pellets, all animals started taking a regular growing diet containing neither antibiotics nor coccidiostatics (**Table 1**). To prevent obesity, feeding was limited to 30–35 g/day of Dry Matter/kg of live weight throughout the experiment. Two different experiments were carried out. The experimental scheme is detailed on **Figure 1**.

**Table 1:** The nutrient composition of the diets.

Feed Composition	Regular diet	High fiber diet
Dry Matter (%)	90.26	93.3
Crude Protein (%)	16.32	2.69
Crude Fat (%)	3.04	1.02
Crude Fiber (%)	14.36	35.05
Crude Ash (%)	7.04	8.40


In experiment 1, in which all animals were fed a regular diet (**Table 1**), rabbits were split in two groups (*n* = 5/treatment); uninfected control (RC: Regular diet Control) and infected with MAP (RI: Regular diet Infected). The RI group was orally challenged 8 weeks after the beginning of the experiment (**Figure 1**) with 2 ml of 2 × 10^8^ cfu/ml of MAP strain K10 for three consecutive days as described previously (Arrazuria et al., 2015a).

In the second experiment, rabbits were also split in two groups (*n* = 5/treatment): (a) uninfected controls taking high fiber diet during the same period as their infected mates (HFC, high fiber control), and (b) twice infected with MAP taking high fiber diet during challenge (HFI, high fiber infected). In this experiment, regular diet was switched to a high fiber diet (**Table 1**) from week 7 to week 11 and from week 23 to week 26 in both groups (**Figure 1**) in order to modulate MAP infection as previously observed (Arrazuria et al., 2015a). The high fiber diet was switched back to the regular diet by replacing 25% of high fiber diet with regular diet weekly. The HFI group was orally challenged at week 8 and 24 with 2 ml of 2 × 10^8^ cfu/ml of MAP strain K10 for three consecutive days as described previously (Arrazuria et al., 2015a).

All four groups were housed in different rooms, with direct contact between animals belonging to the same group, and *ad libitum* water available on accessible containers fixed to the room walls. All animals were euthanized at week 28 of the experiment (36 weeks of age) by intracardiac pentobarbital injection after deep sedation with xylazine (5 mg/kg) and ketamine (35 mg/kg). Animals were exsanguinated and then necropsied.

This study was carried out following European, National, and Regional regulations on animals used in experimentation and other scientific purposes. The procedure was evaluated by the Ethics Committee of the institution (NEIKER-OEBA-2014-0001) and authorized by the Regional Council (BFA-4269).

### Sampling, DNA Extraction, and PCR for MAP Detection

The lymphoid tissues: sacculus rotundus, vermiform appendix, and mesenteric lymph node were excised and stored at -20°C until further analyses.

DNA extraction was performed using DNA Extract-VK (Vacunek S.L, Bizkaia, Spain) following manufacturer’s instructions as described previously (Arrazuria et al., 2015b). Briefly, DNA extraction was carried out on 300 mg of tissue, with a combination of mechanical disruption of the cell walls using glass microspheres (100 μm) agitation and a chemical lysis with SDS and proteinase K. DNA purification was achieved via silica based spin columns (Vacunek S.L, Bizkaia, Spain).

To identify MAP positive samples, DNA was subjected to a real-time multiplex PCR assay targeting the IS*900* and IS*Map02* DNA sequences of MAP (Sevilla et al., 2014). The reaction mixture contained 1x TaqMan Universal PCR Master Mix (Applied Biosystems, Madrid, Spain), 300–400 nM of each primer, 200 nM of each probe, and 5 μl of DNA extract in a final volume of 25 μl. Negative DNA extraction controls, non-template DNA and a positive PCR control were included in each PCR assay. A competitive internal amplification control (IAC) was also included in the PCR reaction. Amplification was carried out in a 7500 Real-Time PCR instrument (Applied Biosystems, Madrid, Spain) and consisted of one denaturation and polymerase activation cycle of 10 min at 95°C, and 45 cycles of denaturation at 95°C for 15 s and annealing/extension at 60°C for 1 min. Results were analyzed using the 7500 System SDS software v. 1.4 (Applied Biosystems). Threshold cycle (*C*_T_) and baseline were automatically determined by the software and verified by visual examination of the threshold line in amplification plots. *C*_T_ values equal or below 38 for both IS*900* and IS*Map02* probes were considered positive, *C*_T_ values over 38 for both targets probe and under 38 for IAC probe were considered negative.

### Sampling and DNA Extraction for 16S rRNA Gene Sequencing

To obtain the samples of cecal content, a small incision about 5 cm long was made in the middle of the cecum and approximately one gram of cecal content was taken and immediately saved in a sterile microtube. The sacculus rotundus was carefully excised and washed with sterile PBS to remove the digesta residues. A fragment was divided and saved immediately in a sterile microtube. All samples were stored in liquid nitrogen until further processing.

The frozen cecal content was defrosted and total DNA was extracted from 150 mg of each sample using a ZR Fecal DNA MiniPrep (Zymo Research, Freiburg, Germany) according to manufacturer’s instructions. This extraction kit includes a bead-beating step for the mechanical lysis of the cells.

The DNA extraction from the sacculus rotundus was carried out with Ultra-Deep Microbiome Prep kit (Molzym, Bremen, Germany) following manufacturer’s instructions. This extraction method allows the enrichment of bacterial DNA and removal of animal and “dead” microbial DNA. Briefly, 0.25 cm^2^ of tissue sample was treated for host cell lysis under chaotropic conditions. The released DNA was then enzymatically degraded, and then the degrading enzymes inactivated. After bacterial cell wall degradation the DNA was extracted and purified via silica based spin columns. DNA concentration and purity of all samples were determined with ND-1000 spectrophotometer (Nanodrop, Wilmington, DE, USA) by measuring the A_260/280_. DNA quality was also evaluated by gel electrophoresis after standard PCR using universal primers pAF (5′-AGA GTT TGA TCC TGG CTC AG-3′) and 530R (5′-CCG CGG CKG CTG GCAC-3′). DNA extracts were stored at -20°C until they were processed.

### Library Construction and MiSeq Illumina Sequencing

The V3–V4 region of 16S rRNA gene was targeted for PCR amplification using a modified F338 and barcoded R806 primers (Caporaso et al., 2012) as described previously (Derakhshani et al., 2016b). Briefly, PCR reaction for each sample was performed in duplicate and contained 1.0 μl of pre-normalized DNA (20 ng/μl), 1.0 μl of each forward and reverse primers (10 μM), 12 μl HPLC grade water (Fisher Scientific, ON, Canada) and 10 μl 5 Prime Hot MasterMix (5 Prime, Inc., Gaithersburg, MD, USA). Reactions consisted of an initial denaturing step at 94°C for 3 min followed by 30 amplification cycles at 94°C for 45 s, 62°C for 60 s, and 72°C for 90 s; finalized by an extension step at 72°C for 10 min in an Eppendorf Mastercycler pro (Eppendorf, Hamburg, Germany). PCR products were then purified using ZR-96 DNA Clean-up Kit (Zymo Research, Irvine, CA, USA) to remove primers, dNTPs and reaction components. The V3–V4 libraries were then generated by pooling 200 ng of each sample, quantified by Picogreen dsDNA (Invitrogen, New York, NY, USA) and diluted to a final concentration of 5 pM, measured by Qubit 2.0 Fluorometer (Life technologies, Ottawa, ON, Canada). In order to improve the unbalanced and biased base composition of the generated 16S rRNA libraries, 15% of PhiX control library was spiked into each amplicon pool. Customized sequencing primers for read1 (5′-TATGGTAATTGTGTGCCAGCMGCCGCGGTAA-3′), read2 (5′-AGTCAGTCAGCCGGACTACHVGGGTWTCTAAT-3′) and index read (5′-ATTAGAWACCCBDGTAGTCCGGCTGACTGACT-3′) were synthesized and purified by polyacrylamide gel electrophoresis (Integrated DNA Technologies, Coralville, IA, USA) and added to the MiSeq Reagent Kit V3 (600-cycle; Illumina, San Diego, CA, USA). The 300 paired-end sequencing reactions were performed on a MiSeq platform (Illumina, San Diego, CA, USA) at the Gut Microbiome and Large Animal Biosecurity Laboratories, Department of Animal Science, University of Manitoba, Canada. The sequencing data were deposited into the Sequence Read Archive (SRA) of NCBI (http://www.ncbi.nlm.nih.gov/sra) and can be accessed via accession number SRR2962702.

### Bioinformatics and Statistical Analyses

The FLASH assembler (Magoč and Salzberg, 2011) was used to merge overlapping paired-end Illumina fastq files. All the sequences with mismatches or ambiguous calls in the overlapping region were discarded. The output fastq file was then analyzed by downstream computational pipelines of the open source software package QIIME version 1.9.0 (Caporaso et al., 2010b). Assembled reads were demultiplexed according to the barcode sequences and exposed to additional quality-filters so that reads with ambiguous calls and those with phred quality scores (Q-scores) below 20 were discarded. Chimeric reads were filtered using UCHIME (Edgar et al., 2011) and sequences were assigned to operational taxonomic units (OTU) using the QIIME implementation of UCLUST (Edgar, 2010) at 97% pairwise identity threshold. Taxonomies assignment of representative OTUs and alignment to Silva reference database (Quast et al., 2013) were performed using PyNAST algorithms (Caporaso et al., 2010a). Phylogenetic trees were built with FastTree 2.1.3 for further comparisons between microbial communities (Price et al., 2010).

Within community diversity (alpha-diversity) was calculated using QIIME scripts. An even depth of 24,500 and 12,400 sequences per sample was used for calculation of species richness (Chao1; Chao, 1984) and diversity indices (Shannon and Simpson) for the cecal content and sacculus rotundus, respectively. Alpha rarefaction curves were generated using Chao1 estimator of species richness (Chao, 1984) with 10 sampling repetitions at each sampling depth. UNIVARIATE procedure of SAS (SAS 9.3, SAS Institute Inc., Cary, NC, USA) was used to test the normality of residuals for alpha-diversity indices and the average of bacterial phyla. A logarithmic transformation was used to normalize the data when necessary. Comparisons between groups were performed using Student’s *t*-test.

The diversity between animals and treatments (beta-diversity) was compared using weighted and unweighted UniFrac distances (Lozupone and Knight, 2005) based on phylogenetic differences. Principal coordinate analysis (PCoA) was applied on resulting distance matrices to generate two-dimensional plots using PRIMER V6 software (Warwick and Clarke, 2006) and permutational multivariate analysis of variance (PERMANOVA; Anderson, 2005) was used to calculate *p*-values and test for differences between microbial communities.

Microbial community between infected and non-infected animals was analyzed through orthogonal projection to latent structures discriminant analysis (OPLS-DA) using the SIMCA 14 software suite (Umetrics, Malmö, Sweden). The OPLS-DA is an extension of PLS-DA, featuring an integrated orthogonal signal correction (OSC) filter to remove variability not relevant to class separation (Trygg and Wold, 2002). In OPLS-DA a regression model is calculated between the multivariate data and a response variable that only contains class information. The OPLS-DA models were fitted with one predictive and two orthogonal components (1 + 2). The quality of the models was evaluated with *R*^2^*Y* and *Q*^2^, indicating the percent of variation of the training set explained by the *Y*-predictive components (infection status) and the cross-validated predicted variation, respectively. An R^2^*Y*-value close to 1 (explained variation) and *Q*^2^ value larger than 0.5 is indicative of a good model with good predictability (Trygg and Wold, 2002).

For all analyses, a *p*-value of <0.05 was considered to be statistically significant and <0.1 was considered as a trend.

## Results

### Determination of MAP Infection

*Mycobacterium avium* subspecies *paratuberculosis* infection was confirmed following detection of MAP by PCR in at least one of the three lymphoid tissues (**Table 2**). All non-challenged animals yielded MAP negative PCR results, whereas all challenged group animals except one belonging to the RI group, were positive for at least one lymphoid tissue. Samples from the animal that rendered the negative PCR result were removed from all downstream analyses, and as such, only animals that presented at least one tissue positive in MAP PCR were included in analyses in their respective infected groups.

**Table 2:** *Mycobacterium avium* subspecies *paratuberculosis* (MAP) PCR results in the infected animals.

		PCR results for indicated site		
		
Group	Animal ID	SR	VA	MLN
RI	16	-	-	-
	17	+	-	-
	18	-	-	+
	19	-	-	+
	20	+	-	+
HFI	11	-	-	+
	12	+	+	+
	13	-	-	+
	14	-	+	+
	15	-	-	+



*RI, regular diet infected; HFI, high fiber diet infected; SR, sacculus rotundus; VA, vermiform appendix; MLN, mesenteric lymph node.*

### Cecal Content Microbiota

**FIGURE 2:**
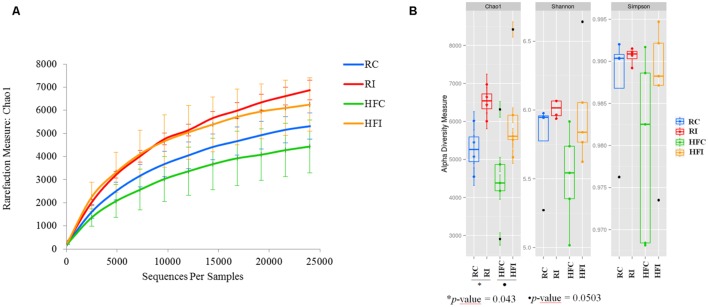
**Cecal content diversity analysis.** **(A)** Cecal content microbiota rarefaction curve generated using Chao1 richness estimator. Samples have been rarified at an even depth of 24,500 sequences per sample. Error bars indicate the 95% confidence intervals. **(B)** Cecal content alpha-diversity; richness (Chao1) and diversity (Shannon and Simpson) indices box plot.

Following quality control and removal of chimeric reads, an average of 40,986 (*SD* = 12,744) high quality sequences were obtained for cecal content samples and used for downstream analyses. Analyses of microbial communities revealed differences in richness (Chao1) between infected and control animals (**Figure 2A**). The RI animals presented higher richness values than RC (6873 ± 481 vs. 5313 ± 651; *p* = 0.0043; **Figure 2B**). An increasing trend was also observed in the Chao1 richness estimates of HFI compared to HFC group (6274 ± 1299 vs. 4434 ± 1273; *p* = 0.0503). However, the Shannon and Simpson indices were not significantly different between infected and control animals in any of the diet groups.

**FIGURE 3:**
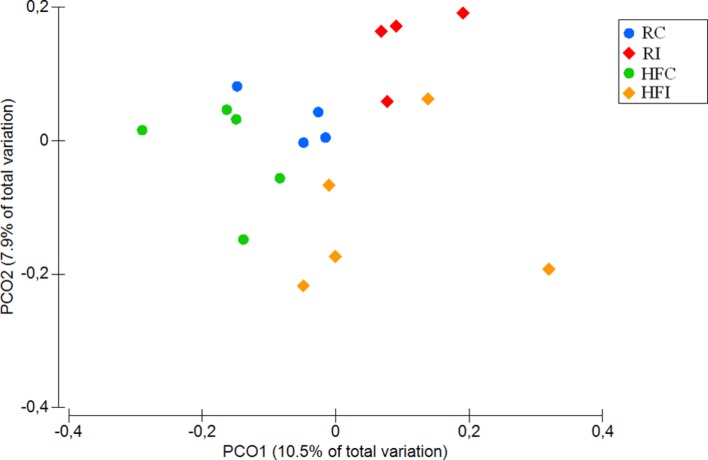
**Cecal content principal coordinates analysis (PCoA) of unweighted UniFrac distances.** Significant *p*-value = 0.01 for Infected vs. non-infected comparison was obtained from PERMANOVA.

The beta-diversity analysis of unweighted UniFrac distances revealed distinct clustering pattern for the cecal content microbiota of MAP infected and control animals (*p* = 0.01; **Figure 3**). However, when the weighted UniFrac distances were analyzed, no significant differences were observed between the microbiota composition of infected and non-infected animals (*p* = 0.2; **Supplementary Figure S1**).

**FIGURE 4:**
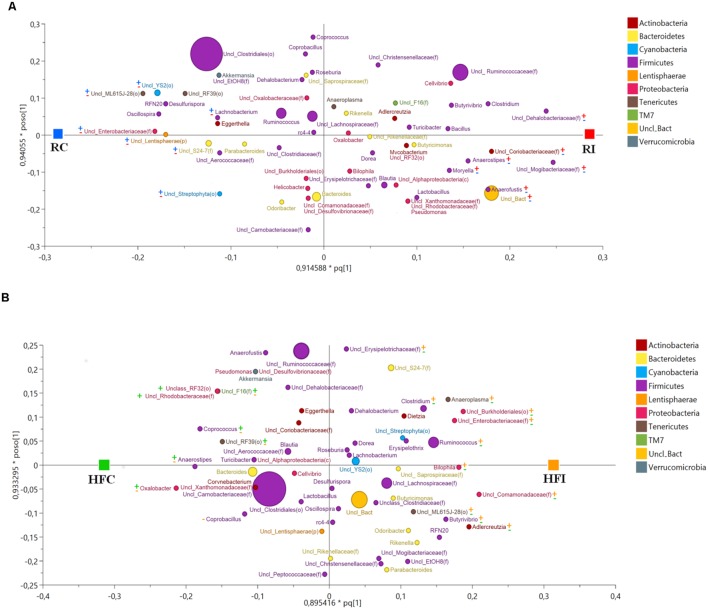
**Cecal content microbiota orthogonal projection to latent structures discriminant analysis (OPLS-DA) scatter plot.** The size of the spheres represents abundance. **(A)** OPLS-DA Scatter Plot for cecal content samples of animals fed with regular diet (*R*^2^*Y* = 1; *Q*^2^ = 0.678). The taxa with significant positive association with MAP infected animals are labeled with (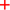) and with negative association with MAP infected animal with (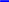). The taxa with significant positive association with control animals are labeled with (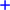) and with negative association with control animals with (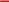). **(B)** OPLS-DA scatter plot for cecal content samples of animals fed with high fiber diet (*R*^2^*Y* = 0.988; *Q*^2^*Y* = 0.733). The taxa with significant positive association with MAP infected animals are labeled with (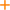) and with negative association with MAP infected animal with (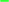). The taxa with significant positive association with control animals are labeled with (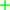) and with negative association with control animals with (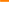).

Alignment of OTUs at 97% similarity threshold against Silva database resulted in identification of seven bacterial phyla and 51 bacterial genera in the cecal content (**Table 3**, **Figure 4**). While majority of OTUs were identified at the genus level, some were only classified at the phylum, class, order, or family. The cecal content microbiota was dominated by members of phylum Firmicutes followed by members of phylum Bacteroidetes. Among all phyla, only the abundance of Proteobacteria significantly declined following MAP infection in animals fed high fiber diet (*p* = 0.019).

**Table 3:** Average percentages of bacteria phyla in the cecal content.

Phyla	Mean values, %, under indicated conditions (SEM)				*p*-values	
		
	Treatments				RC vs. RI	HFC vs. HFI
			
	RC	RI	HFC	HFI		
Firmicutes	71.01 (3.59)	69.05 (2.16)	64.75 (0.56)	64.99 (0.59)	0.725	0.918
Bacteroidetes	13.73 (4.70)	12.21 (1.70)	16.86 (1.36)	14.16 (1.37)	0.755	0.554
Cyanobacteria	4.23 (1.00)	1.72 (0.82)	4.19 (1.03)	5.18 (1.08)	0.118	0.731
Actinobacteria	0.39 (0.07)	0.56 (0.05)	0.51 (0.01)	0.48 (0.02)	0.100^2^	0.817
Proteobacteria	0.16 (0.10)	0.29 (0.09)	0.07 (0.00)	0.06 (0.00)	0.797	0.019^1^
TM7	0.16 (0.03)	0.26 (0.04)	0.47 (0.02)	0.16 (0.02)	0.853	0.147
Tenericutes	0.43 (0.13)	0.16 (0.05)	0.34 (0.06)	0.78 (0.07)	0.955	0.052^2^
Unassigned	9.89 (0.93)	15.75 (2.61)	12.81 (1.04)	14.18 (1.42)	0.132	0.732


*SEM, standard error of the mean. RC, regular diet control; RI, regular diet infected; HFC, high fiber diet control; HFI, high fiber diet Infected. ^1^*P* < 0.05 was considered significant. ^2^*P* < 0.1 was discussed as trend.*

The OPLS-DA of cecal content bacteria for *Y* variable (infection) yielded a suitable model for microbial comparison between infected and non-infected animals fed the regular (*R*^2^*Y* = 1; *Q*^2^ = 0.678) or the high fiber diets (*R*^2^*Y* = 0.988; *Q*^2^ = 0.733; **Figure 4**).

In RI animals most of the bacteria positively associated with MAP infection belonged to the phylum Firmicutes including family Dehalobacteriaceae, Mogibacteriaceae, Lachnospiraceae (including genera *Anaerostipes* and *Moryella*) and Eubacteriaceae (including genus *Anaerofustis*). In addition, family Coriobacteriaceae (phylum Actinobacteria) was more abundant in RI group than in RC (**Figure 4A**, **Supplementary Figure S2A**). On the other hand bacterial phyla showing negative association with MAP infection while positively related with RC group were more diversified including genus *Lachnobacterium* (phylum Firmicutes); family Enterobacteriaceae (phylum Proteobacteria); family S24-7 (phylum Bacteroidetes); orders YS2 and Streptophyta (phylum Cyanobacteria); order ML615J-28 (phylum Tenericutes), and unclassified members of phylum Lentisphaerae (**Figure 4A**, **Supplementary Figure S2A**).

Animals fed the high fiber diet showed a different pattern of bacteria positively associated with MAP infection (**Figure 4B**, **Supplementary Figure S2B**). Within the phylum Firmicutes, several taxa including genera *Ruminococcus*, *Clostridium*, and *Butyrivibrio*, and family Erysipelotrichaceae were positively associated with HFI. In this group, members of phylum Proteobacteria were more abundant than in RI group. Examples included family Enterobacteriaceae (class Gammaproteobacteria); family Comamonadaceae; order Burkholderiales (class Betaproteobacteria); and genus *Bilophila* (class Deltaproteobacteria). Bacterial lineages belonging to the phylum Actinobacteria (genus *Adlercreutzia*) and phylum Tenericutes (genus *Anaeroplasma* and family ML615J-28) were also more abundant in the HFI group compared to HFC.

The bacterial taxa showing negative association with MAP infection in animals fed high fiber diet and therefore more abundant in the HFC group, were different from those described in the RC group. These included genera *Coprococcus*, *Anaerostipes*, and *Coprobacillus* (phylum Firmicutes); genus *Oxalobacter*, family Rhodobacteraceae, and order RF32 (phylum Proteobacteria); order RF39 (phylum Tenericutes); and family F16 (phylum TM7; **Figure 4B**, **Supplementary Figure S2B**).

### Sacculus Rotundus Microbiota

**FIGURE 5:**
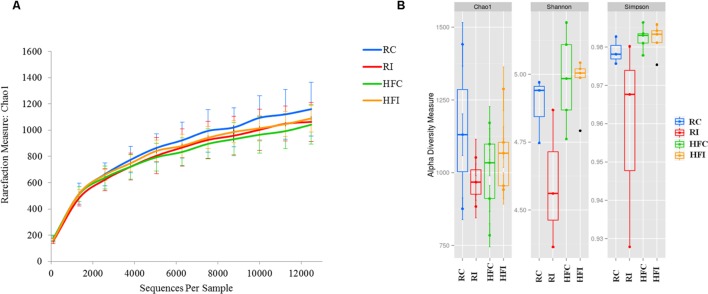
**Sacculus rotundus diversity analysis.** **(A)** Sacculus rotundus rarefaction curve generated using Chao1 richness estimator. Samples have been rarified at an even depth of 12,400 sequences per sample. Error bars indicate the 95% confidence intervals. **(B)** Sacculus rotundus alpha-diversity; richness (Chao1) and diversity (Shannon and Simpson) indices box plot.

**FIGURE 6:**
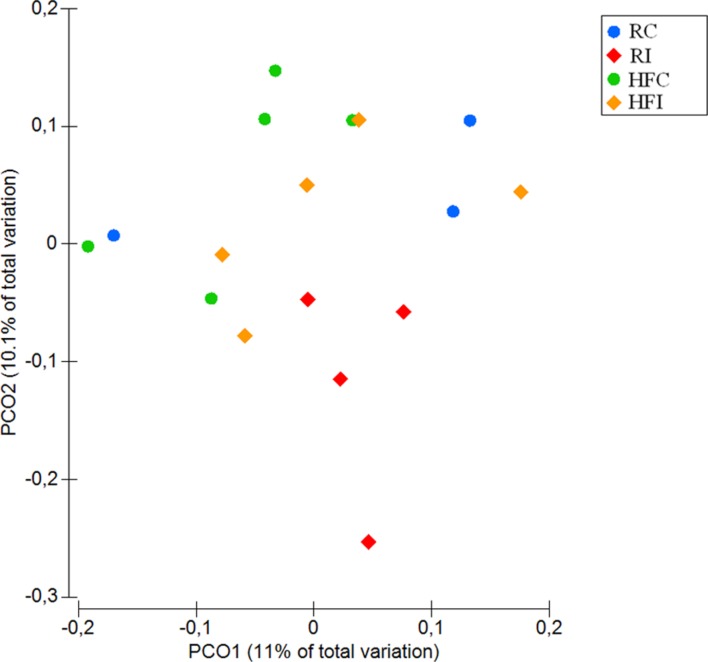
**Principal coordinates analysis (PCoA) of unweighted UniFrac distances of samples from sacculus rotundus.** Significant *p*-value = 0.032 for Infected vs. non-infected comparison was obtained from PERMANOVA.

**FIGURE 7:**
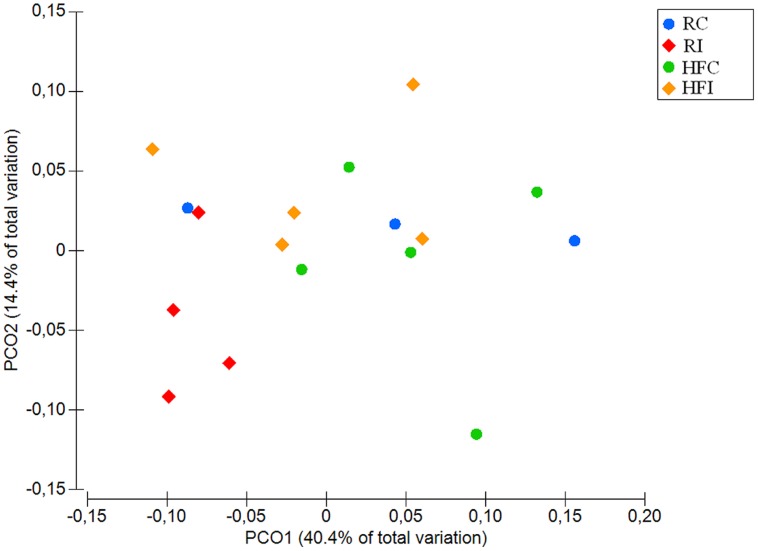
**Principal coordinates analysis (PCoA) of weighted UniFrac distances of samples from sacculus rotundus.** Significant *p*-value = 0.048 for Infected vs. non-infected comparison was obtained from PERMANOVA.

An average of 26,900 (*SD* = 10,284) of high quality sequences were obtained from sacculus rotundus samples. There were no significant differences in richness (Chao1 index), or other alpha-diversity indices (Shannon and Simpson) when infected and non-infected animals were compared (**Figure 5**). The beta-diversity analysis of UniFrac distances revealed differences between infected and non-infected animals in both weighted (*p* = 0.048) and weighted (*p* = 0.032) measures (**Figures 6** and **7**). In this lymphoid tissue, 7 bacteria phyla and 63 bacteria genera were identified.

**FIGURE 8:**
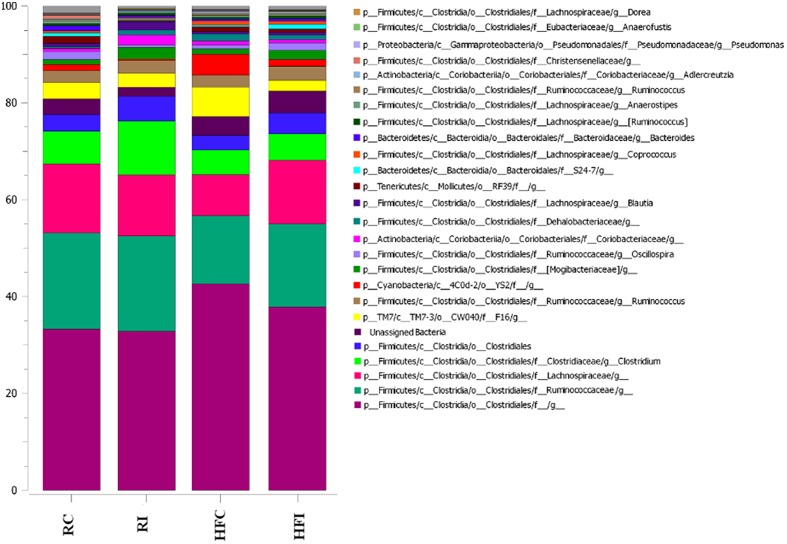
**Stack-bar of the most abundant OTUs in sacculus rotundus.** Vertical bars demonstrate the proportion (%) of the most abundant bacterial taxa (>0.1% of community). Taxonomic classification of each taxon is identified at phylum (p_), class (c_), order (o_), family (f_), and genus (g_) level.

Similar to the cecal content, sacculus rotundus microbiota was mainly composed of phylum Firmicutes (**Table 4**). In contrast, phylum TM7 was the second most abundant phylum in the sacculus rotundus ranging from 2.3 to 5.9% of community, while its relative abundance ranged from 0.16 to 0.47% in the cecal content. The only phylum that significantly shifted with infection was Bacteroidetes, with declining abundance in MAP infected rabbits fed the regular diet. The composition of the OTUs at the genus level in the four groups is presented in **Figure 8**.

**Table 4:** Average percentage of bacteria phyla in the sacculus rotundus samples.

Phyla	Mean values, %, under indicated conditions (SEM)				*p*-values	
		
	Treatments				RC vs. RI	HFC vs. HFI
			
	RC	RI	HFC	HFI		
Firmicutes	89.28 (5.37)	89.55 (1.61)	81.78 (3.56)	87.86 (1.80)	0.414	0.178
TM7	2.71 (1.74)	4.27 (1.19)	5.97 (1.65)	2.30 (0.69)	0.821	0.065^2^
Bacteroidetes	2.13 (0.11)	1.49 (0.25)	0.93 (0.27)	1.75 (0.75)	0.014^1^	0.261
Actinobacteria	0.95 (0.35)	2.14 (0.64)	1.17 (0.20)	0.99 (0.19)	0.150	0.378
Tenericutes	0.83 (1.11)	0.27 (0.09)	0.95 (0.39)	0.79 (0.20)	0.398	0.504
Cyanobacteria	0.66 (1.11)	0.20 (0.10)	5.14 (1.95)	1.36 (0.54)	0.467	0.212
Proteobacteria	0.49 (0.64)	0.17 (0.03)	0.40 (0.15)	0.17 (0.04)	0.428	0.136
Unassigned	2.97 (0.82)	1.91 (0.08)	3.66 (0.60)	4.76 (1.16)	0.242	0.620


*SEM, standard error of the mean. RC, regular diet control; RI, regular diet infected; HFC, high fiber diet control; HFI, high fiber diet infected. ^1^*P* < 0.05 was considered significant. ^2^*P* < 0.1 was discussed as trend.*

**FIGURE 9:**
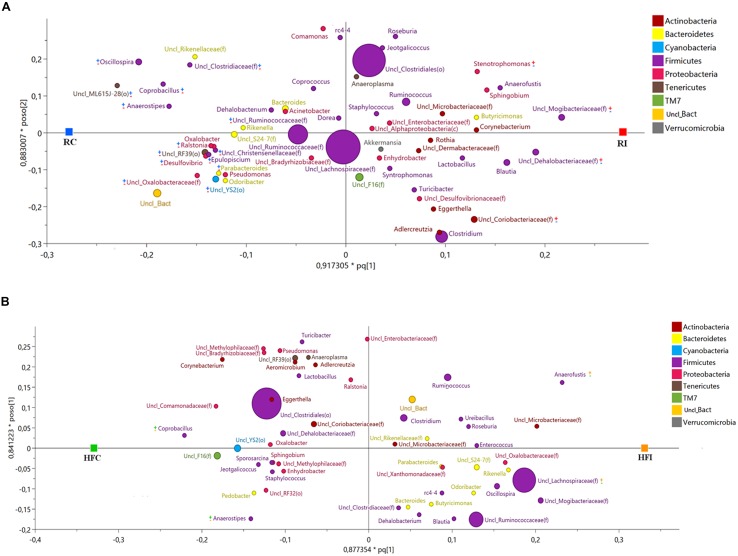
**Sacculus rotundus microbiota orthogonal projection to latent structures discriminant analysis (OPLS-DA) scatter Plot.** The size of the spheres represents abundance. **(A)** OPLS-DA scatter plot for sacculus rotundus samples of animals fed with regular diet (*R*^2^*Y* = 0.995; *Q*^2^ = 0.765). The taxa with significant positive association with MAP infected animals are labeled with (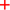) and with negative association with MAP infected animal with (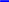). The taxa with significant positive association with control animals are labeled with (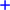) and with negative association with control animals with (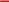). **(B)** OPLS-DA scatter plot for sacculus rotundus samples of animals fed with high fiber diet (*R*^2^*Y* = 0.921; *Q*^2^ = 0.511). The taxa with significant positive association with MAP infected animals are labeled with (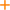) and with negative association with MAP infected animal with (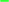). The taxa with significant positive association with control animals are labeled with (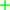) and with negative association with control animals with (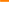).

As in the cecal content, the OPLS-DA analysis of bacterial community in sacculus rotundus for *Y*-variable (infection) yielded a model with high goodness of fit (*R*^2^*Y*) and predicted value (*Q*^2^) both for animals fed with regular diet (*R*^2^*Y* = 0.995, *Q*^2^ = 0.765) and high fiber diet (*R*^2^*Y* = 0.921, *Q*^2^ = 0.511; **Figure 9**).

Several bacterial taxa were positively associated with MAP infection and therefore more abundant in the RI compared to the RC group. This included the families Mogibacteriaceae, Dehalobacteriaceae, and Coriobacteriaceae and the genus *Stenotrophomonas*. A large number of taxa had negative association with MAP infection and therefore were overrepresented in the RC group comparing to RI one (**Figure 9**, **Supplementary Figures S3A,B**). Among these taxa, three also had negative association with infection in the cecal content and belonged to the phylum Tenericutes (order ML615J-28) and phylum Cyanobacteria (orders Streptophyta and YS2; **Figure 9A**, **Supplementary Figure S3A**).

Among animals fed the high fiber diet, genus *Anaerofustis* and family Lachnospiraceae were more abundant in the HFI group compared to the HFC one and therefore they were positively associated with infection. The two taxa with negative association with infection and therefore more abundant in the HFC group, belonged to the phylum Firmicutes, including families Erysipelotrichaceae (genus *Coprobacillus*) and Lachnospiraceae (genus *Anaerostipes*; **Figure 9B**, **Supplementary Figure S3B**). These two genera were also more abundant in the cecal content of HFC group and thus negatively associated with MAP infection.

## Discussion

In the present work, we attempt to study the impact of MAP infection on gut microbiota composition under two different dietary scenarios. Variations in the microbial community were observed depending on the dietary group and in response to MAP infection.

Cecal microbial community of rabbit has been assessed in recent studies (Bäuerl et al., 2014; Zhu et al., 2015) but to the best of our knowledge, this is the first work describing the microbiota of rabbit sacculus rotundus. This organ unique to rabbits is characterized by an ampoule-like enlargement of the terminal ileum. Due to its location and lymphoid nature it might play a similar role to the ileocecal-valve in ruminants (Besoluk et al., 2006) and can be assumed to bear microbiota with an important role in immune regulation.

Studies using high-throughput sequencing technology to explore the gut microbiota in rabbits are not abundant. These limited studies indicate that GI tract of rabbits is a highly diverse environment (Bäuerl et al., 2014; Zhu et al., 2015). In the present study, we observed that even with an average sequencing depth of 24,700 reads/sample in the cecal content and 12,400 reads/sample in the sacculus rotundus, rarefaction curves did not reach a plateau phase indicating that the ecosystem is highly diverse. These findings further support previous observations on high microbiota diversity in rabbit cecal content (Bäuerl et al., 2014; Zhu et al., 2015) and feces (Eshar and Weese, 2014; Zeng et al., 2015).

Microbiota richness, evenness and diversity have been reported as characteristic features determining state of health or disease. A decreased in richness and diversity indices has been reported in CD (Manichanh et al., 2006; Dicksved et al., 2008; Walker et al., 2011) and Type 1 diabetes patients (Giongo et al., 2011), whereas bacterial vaginosis (Ling et al., 2010) and helminthic parasitosis (Lee et al., 2014) have been associated with increased richness. In our study, the alpha-diversity indices in sacculus rotundus microbiota, did not differ between infected and non-infected animals. However, in the cecal content, we found higher values of richness (chao1 index) in infected animals whereas the abundance-based diversity measures (Shannon and Simpson indices) did not vary between infected and non-infected animals. It must be taken into account that cecal content DNA extraction did not allow removing DNA from dead bacteria whereas, the extraction method used for sacculus rotundus did, and this could have influenced results.

The diversity analyses between animals (beta-diversity) revealed that the microbial composition between infected and non-infected animals varied in the cecal content and sacculus rotundus in response to MAP infection. In the cecal content, the significant differences were observed only in unweighted UniFrac distances, suggesting that MAP infection has an impact on the presence and/or absence of certain members of the microbial community rather than on the abundance of these. Due to rabbits’ practice of cecotrophy, the composition of the microbial community of the cecal content may play a role in the modulation of nutrient absorption. In contrast, in sacculus rotundus, we observed changes in beta-diversity using both weighted and unweighted UniFrac distances, suggesting that MAP infection both impacted the presence and/or absence of certain taxa as well as their abundance. These observed changes may play a role in inflammation as demonstrated in previous reports where specific microbial compositions in rabbit’s lymphoid tissue have been seen to induce inflammation (Shanmugam et al., 2005).

It was not surprising that rabbit cecal content was dominated by the phylum Firmicutes as reported in recent studies (Bäuerl et al., 2014; Zhu et al., 2015). This phylum contains some of the major cellulolytic and fibrolytic organisms (Daly et al., 2001) that can be also found in other hindgut fermenters, such as horses (Costa et al., 2012).

At the phylum level, we only observed significant differences in the phyla Proteobacteria and Bacteroidetes. The phylum Proteobacteria decreased in the cecal content of HFI group compared with HFC. However, in the animals fed with high fiber diet there were more bacteria belonging to this phylum positively associated with MAP infection than in animals fed with regular diet. Proteobacteria are considered to be minor and opportunistic components of the gut ecosystem. The proportion of this phylum increases in both IBD patients (Gophna et al., 2006) and animal models of IBD (Munyaka et al., 2016) suggesting these bacteria may play a role in pathogenesis of IBD (Mukhopadhya et al., 2012).

In our study, the phylum Bacteroidetes was decreased in the sacculus rotundus of RI compared with RC. This phylum is reported to decrease in experimentally MAP-infected calves (Derakhshani et al., 2016a) and in CD patients (Gophna et al., 2006). The phylum Bacteroidetes has a major role in degrading complex polysaccharides (Terrapon and Henrissat, 2014) that are normally present in high fiber diets. Studies have shown that high fiber based diets promote the abundance of Bacteroidetes in rabbits gut (Nicodemus et al., 2005; Zhu et al., 2015). In our previous study, we reported that a shift to a high fiber diet during MAP challenge may decelerate the progression of infection (Arrazuria et al., 2015a). The increase of Bacteroidetes may be in part responsible for this finding.

Genus *Mycobacterium* was only detected in one cecal content sample from an animal belonging to the infected group fed the regular diet (**Figure 4A**, **Supplementary Figure S2A**). Since the classification was at the genus level we cannot confirm that it is MAP. In slaughtered rabbits, detection of other members of *Mycobacterium avium* complex has been reported in lymphoid tissue, although no mycobacteria were found in the cecal content (Arrazuria et al., 2015b). The differences in MAP detection in sacculus rotundus by PCR (Arrazuria et al., 2015b) and sequencing could be due to the standardization of amount of DNA for sequencing, the choice of primers used (universal bacterial primers in case of sequencing vs. MAP specific primers for PCR) or because the relative abundance of MAP in infection is not too high since the disease is of chronic nature.

We used an OPLS-DA model to determine which bacteria were positively or negatively associated with MAP infection. Using this approach, we observed that three families, Coriobacteriaceae, Dehalobacteriaceae, and Mogibacteriaceae were positively associated with MAP infection in both sacculus rotundus and the cecal content of the RI group. Some members of the family Coriobacteriaceae have been found in vaginosis, bacteriemia, and periodontitis, being considered pathobionts (Clavel et al., 2014). In the context of PTB, the increase of this family of pathobionts may have an implication in disease progression. Derakhshani et al. (2016a) reported that the family Mogibacteriaceae was the main overrepresented bacteria in the ileum mucosa of MAP infected calves. Unfortunately there is a lack of knowledge on the role that this family could play in gut inflammation.

The genus *Anaerostipes* was found to be more abundant in the cecal content of the RI group. It was also more abundant in sacculus rotundus of the RC and in cecal content and sacculus rotundus of HFC animals. The genus *Anaerostipes* can utilize lactate to produce butyrate (Sato et al., 2008), which can suppress pro-inflammatory cytokine production by intestinal macrophages (Chang et al., 2014) and it is beneficial to colonic health providing protection against colitis (Segain et al., 2000). Also, this genus was found in significant lower abundance in smoker CD patients compared to non-smokers (Morgan et al., 2012). These findings suggest that this genus may play an important role in GI health.

The genus *Stenotrophomonas* was overrepresented in sacculus rotundus of RI. It has been described in CD, and has been recognized in many clinical specimens and inflammatory conditions like pneumonia, bacteremia, and urinary tract infection (Denton and Kerr, 1998; Knösel et al., 2009). This species also appears to be increased in mice females infected with MAP (Karunasena et al., 2014). These findings are in agreement with our results.

The genus *Coprobacillus* was more abundant in the control groups (sacculus rotundus of RC and in sacculus rotundus and cecal content of HFC). This genus has been found to be beneficial through maintaining intestinal stability and conferring resistance against *Clostridium difficile* colonization (Stein et al., 2013).

In the present study, sacculus rotundus and cecal content of the RC group showed higher levels of two orders that belong to the phylum Cyanobacteria (orders Streptophyta and YS2). Since Cyanobacteria has been detected in the GI tract of human and other mammals (Ley et al., 2008) the efforts to better characterize the function of members of this phylum are increasing (Rienzi et al., 2013; Soo et al., 2014). Streptophyta, which derive from chloroplasts within plant matter has been detected in high proportion in stool from a patient with resistant tuberculosis treated with broad-spectrum antibiotics (Dubourg et al., 2013), suggesting that the critical reduction of bacterial sequences detected had permitted the amplification of DNA from green plant foods absorbed by the patient. A recent genomic study showed that YS2 does not have photosynthetic ability. Metabolic analysis demonstrated that YS2 has many special functions including obligate anaerobic fermentation, syntrophic H2-production, nitrogen fixation, and synthesis of vitamin B and K (Rienzi et al., 2013). The infection may lead to the establishment of an ecosystem that is not favorable for this order and it could support the pathogenesis of the disease.

As shown in **Figures 4** and **9**, we found additional taxa that show positive or negative relation with MAP infection only under specific dietary regimens. Diets containing different amounts of fiber are likely to differentially modulate the composition of the intestinal microbiota (Serino et al., 2011; Zhu et al., 2015). We cannot rule out that some of the detected microbes were taken up with the diet. DNA from food adsorbed by the host has been reported as mentioned previously (Dubourg et al., 2013). Microbes taken up with food may succeed to survive, especially in the high fiber diet groups where the diet shift itself could have favored their survival. Finally, we cannot, speculate if the associated taxa with MAP-infection, only observed in animals fed the high fiber diet, are consequence of the short diet change, or on the contrary, are the result of MAP reinfection.

## Conclusion

The MAP infection and dietary changes shift microbiota of the cecal content and the sacculus rotundus of rabbits. While some taxa seem to be positively associated with MAP infection in different sites or conditions, there are others that show opposite patterns. Knowing the composition of the microbial community alone does not lead to an understanding of its function. Therefore, it would be helpful to confirm and complete the results presented in the present study with a shotgun metagenomic or metatranscriptomics approach that could help to better understand the role of microbiota composition and function. Further research is required to understand the pathogenesis of MAP associated to chronic intestinal inflammation and the role that intestinal microbiota plays during this process.

## Author Contributions

RJ and NE conceived and designed the experiment. RA and NE conducted the animal experiment. RA and HD performed lab analyses. RA, HD, and EK developed the bioinformatics and statistical models. RA and HD analyzed the data. All authors drafted the manuscript. All authors carefully read and approved the final version of the manuscript.

## Conflict of Interest Statement

The authors declare that the research was conducted in the absence of any commercial or financial relationships that could be construed as a potential conflict of interest.

## Supplementary Material

**FIGURE S1 | Cecal content principal coordinates analysis (PCoA) of weighted UniFrac distances.** PERMANOVA analysis showed no significant differences between Infected vs. non-infected animals *p*-value = 0.2.

**FIGURE S2 | Cecal content OPLS-DA coefficient plot; bars with negative values indicating bacteria that are significantly lower in MAP infected animals and bars with positive value indicating bacteria which are significantly higher in MAP infected animals than in control animals.** Only bacteria with a confidence interval that did not cross the zero line were significantly changed ^∗^*p* < 0.05. **(A)** OPLS-DA coefficient plot for samples of sacculus rotundus from animals infected and fed with regular diet (*R*^2^*Y* = 1; *Q*^2^ = 0.678). **(B)** OPLS-DA coefficient plot for samples of sacculus rotundus from animals infected and fed high fiber diet (*R*^2^*Y* = 0.988; *Q*^2^*Y* = 0.733).

**FIGURE S3 | Sacculus rotundus OPLS-DA coefficient plot; bars with negative values indicating bacteria that are significantly lower in MAP infected animals and bars with positive value indicating bacteria which are significantly higher in MAP infected animals than in control animals.** Only bacteria with a confidence interval that did not cross the zero line were significantly changed ^∗^*p* < 0.05. **(A)** OPLS-DA coefficient plot for samples of sacculus rotundus from animals infected and fed with regular diet (*R*^2^*Y* = 0.995; *Q*^2^ = 0.765). **(B)** OPLS-DA coefficient plot for samples of sacculus rotundus from animals infected and fed high fiber diet (*R*^2^*Y* = 0.921; *Q*^2^ = 0.511).
