# Supplementary figures and images for: Mycobacterium avium Subspecies paratuberculosis Infection Modifies Gut Microbiota under Different Dietary Conditions in a Rabbit Model
Source: Front Microbiol. 2016 Mar 31;7:446. doi: 10.3389/fmicb.2016.00446 (PMC4815054; doi:10.3389/fmicb.2016.00446)

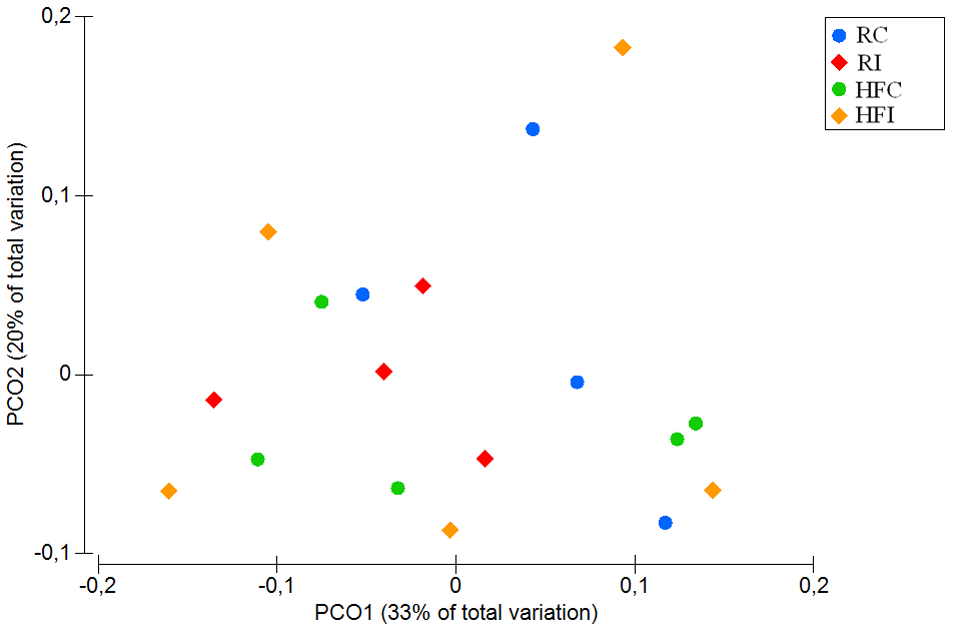

Supplement: Supplementary file 1 [file Image_1.TIF]

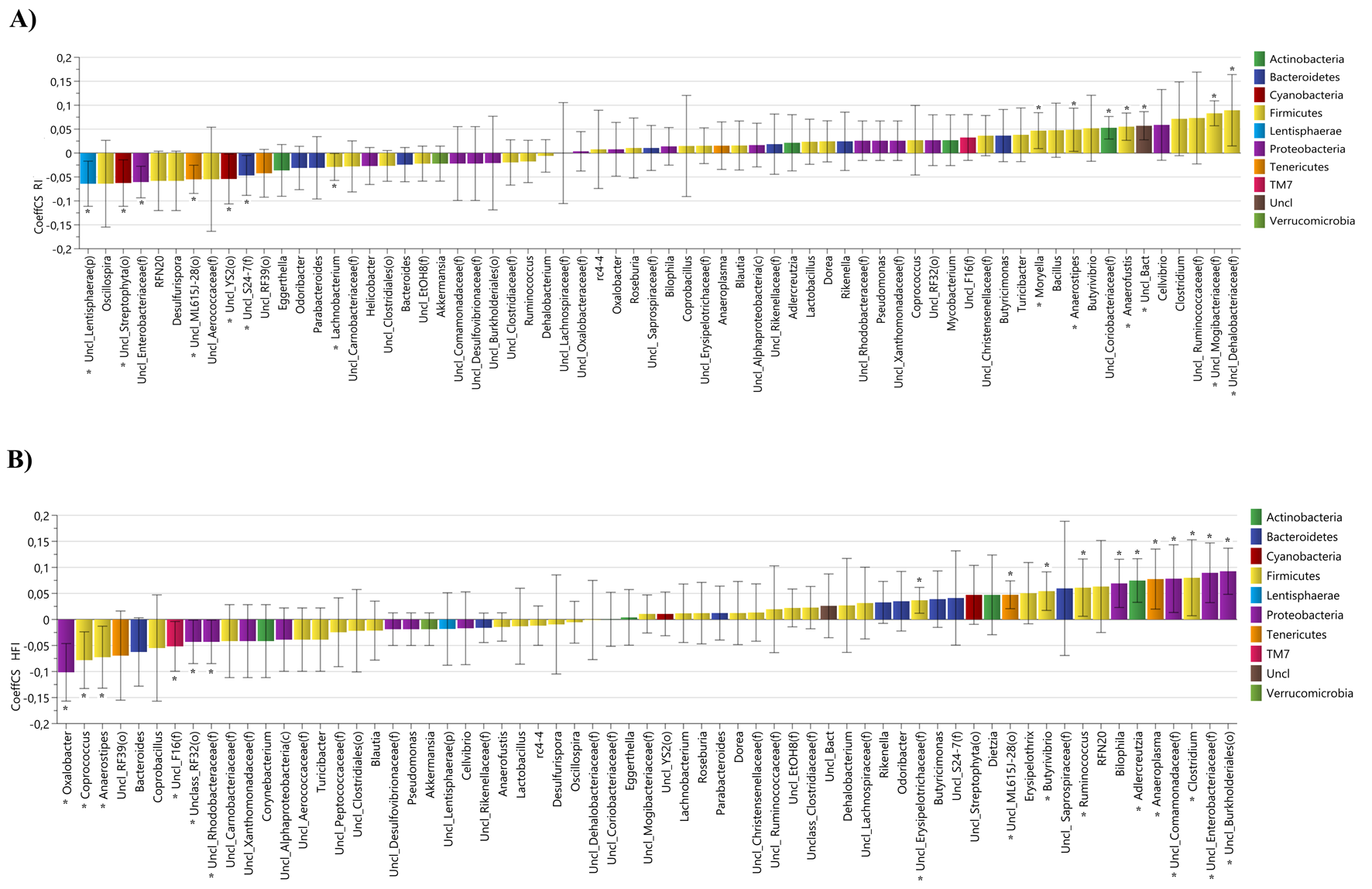

Supplement: Supplementary file 2 [file Image_2.TIF]

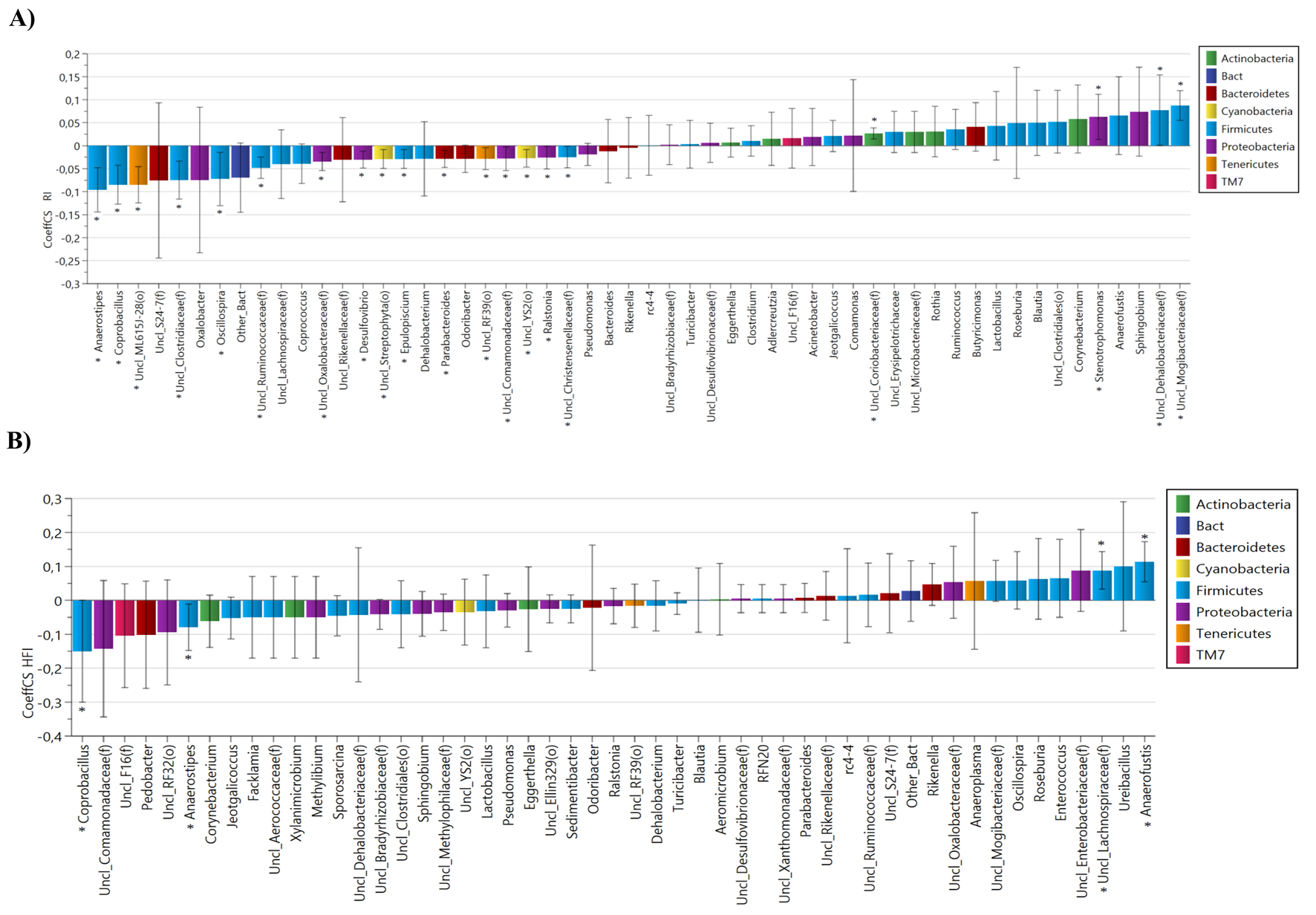

Supplement: Supplementary file 3 [file Image_3.TIF]
